# Contamination and Probabilistic Ecological–Health Risk of Heavy Metal(loid)s in Urban Topsoil of Mianyang, SW China

**DOI:** 10.3390/ijerph192215126

**Published:** 2022-11-16

**Authors:** Huaming Du, Xinwei Lu

**Affiliations:** 1 School of Resource and Environment Engineering, Mianyang Normal University, Mianyang 621000, China; 2 Department of Environmental Science, School of Geography and Tourism, Shaanxi Normal University, Xi’an 710119, China

**Keywords:** heavy metal(loid), hot spot analysis, contamination assessment, probabilistic risk assessment, Monte Carlo simulation, topsoil

## Abstract

Heavy metal(loid) (HM) pollution in urban topsoil seriously endangers the health of urban residents and urban sustainable development. Compared with large cities, the research on the HM pollution of topsoil in emerging medium-sized industrial cities is quite limited. This study focuses on topsoil HM contamination in Mianyang, which is a representative moderate emerging industrial city in Southwest China. The results indicate that Ba, Cr, Cu, and Zn in the samples were much higher than their background values. The hot spots of Ba, As, Cu, Pb, Co, Cr, and Zn showed an obvious enrichment trend. The potential ecological risk of HMs showed a low ecological risk, which was mainly caused by As. The investigated HMs presented no significant non-carcinogenic hazard to local adult residents, but there were three sampling sites which presented a non-carcinogenic hazard to children; the carcinogenic risks of As, Cr, Co, and Ni were acceptable. In this study, a mixed source of industry and traffic was identified to be the priority anthropogenic source, and Cr and As were identified as the priority elements for further risk control. The findings of our study could be beneficial to decision-makers with regard to taking appropriate measures to control and reduce HM pollution in the Mianyang urban area.

## 1. Introduction

Cities are the main spaces for human activities and are places of concentrated living. Urban topsoil, an essential part of the urban ecosystem, is not only the sink of urban pollutants but also the secondary pollution source of urban water and the atmosphere. Rapid industrialization and urbanization have promoted the sustained growth of the economy and the improvement of people’s quality of life. Simultaneously, a lot of pollutants such as heavy metal(loid)s (HMs) enter the urban soil environment [1,2]. HMs are a kind of toxic pollutants that are easy to accumulate and difficult to degrade. They are widely distributed in natural environments and have the characteristics of lasting long-term, concealment, and non-degradability [3,4]. With the advancement of urbanization, industrial waste [5], motor vehicle exhausts [6], and domestic waste [7] increase the pollution by HMs in urban soil. HMs in urban soil may be highly concentrated in the human body through direct ingestion, inhalation, and skin contact [8], leading to acute and chronic poisoning and many kinds of physical damage, which are threatening to human health [9]. Some reports show that when HMs in the soil enter the human body, they have non-carcinogenic effects on the human nervous system [10] and immune function [3]. However, there are studies which report that some HMs are also highly carcinogenic [11]. HM pollution in urban topsoil is directly related to the health of urban populations, which has attracted much attention. Scholars from different countries have studied HM pollution in the urban topsoil of different cities [12,13,14].

In China, research reports about the HM pollution of urban topsoil and health risk assessments are increasing. Nevertheless, studies about the HM pollution of urban topsoil in China are mainly concentrated on large cities, such as Beijing [15], Shanghai [14], Ningbo [5], Changchun [16], and moderately heavy industrial cities such as Baotou [17], Anshan [18], and Karamay [19]. There is little research on emerging industrial cities. Emerging industrial cities are cities with electronics, automobiles, precision instruments, equipment manufacturing, bio-pharmaceuticals, new materials, and new energies as the leading industries. They are cities with a high concentration of capital stock, assets, and high-level technologies. Their industrial productions are characterized by high-value-added products. Industrial activities not only increase the consumption of energy resources but also continuously generate industrial waste in the production process, which is not conducive to the healthy development of urban environments [20]. In particular, the use of HMs and chemical materials in the production of electronics, automobiles, and mechanical products leads them to enter the soil directly or indirectly during production, recovery, and treatment, which may cause HM pollution in urban topsoil [21]. HM pollution seriously threatens the urban environment and human health; therefore, it is important to research HM pollution in the topsoil of emerging industrial cities.

As an emerging industrialized city and an important node city for the comprehensive development of the Chengdu–Chongqing urban agglomeration [22], Mianyang City has formed a new industrial economic system with medicine, electronics, the chemical industry, machinery, new energies, and automobile manufacturing as the main components in recent years with the acceleration of urbanization [22]. At the same time, it has also brought great challenges to environmental protection [20]. The results of spatial distribution and source apportionment indicate that local industrial production and traffic emissions caused the spatial heterogeneity of HMs in the urban topsoil of Mianyang [20]. However, the pollution levels and ecological–health risks of HMs in the urban topsoil of Mianyang are still unclear. The primary aims of this study were to: (1) explore the content and hot spots of some widely concerned HMs (Ba, As, Cr, Co, Cu, Pb, Mn, Ni, Zn, and V) in the urban topsoil of Mianyang; (2) estimate the contamination and ecological risk of the HMs using *I_geo_* and *RI* combing with Monte Carlo simulation (MCS); and (3) evaluate the probabilistic health risk of the HMs in the topsoil to local residents using the USEPA health exposure risk model and MCS. In this study, MCS was used to identify the priority sources and the priority pollutants; the results of this study are crucial for local environmental sustainability and public health.

## 2. Materials and Methods

### 2.1. Study Area

Mianyang City (30°42′ N to 33°03′ N latitude, 103°45′ E to 105°43′ E longitude) is distributed in the northwestern area of Sichuan Basin and the upper and middle reaches of Fujiang River [23]. Cinnamon and loess are the dominating soil types in Mianyang. The landform is dominated by low mountains, hills, and plains and is generally inclined from northwest to southeast, with an elevation of 275–4888 m. Mianyang comprises a total area of 20,248 km^2^ and a built-up urban area of 163 km^2^ [24]. It has a northern, subtropical, mountainous, humid, monsoon climate, with an annual average temperature of 15.4 to 18.1 °C and an annual precipitation of 545.5 to 1699.7 mm. The prevailing wind is southeast and north. The survey region is situated in the urban area of Mianyang, which is a highly urbanized area with an urban population of 1.75 million in 2020 [24]. The gross domestic product of Mianyang reached CNY 1592 billion in 2020, and there was an excess of 1.01 × 10^6^ motor vehicles in Mianyang urban area in 2020 [24]. With the rapid expansion of built-up areas in Mianyang, the city has gradually become a major area for education, residence, commerce, and industry [20]. The eastern part of the urban region is an industrial zone, the region close to the river junction is the historic center district with intensive residential and commercial activities, the northwest is a science and technology industrial zone, the western part is a high-tech industrial zone, and the central part is an educational and residential zone [20].

### 2.2. Sampling and Monitoring

**Figure 1:**
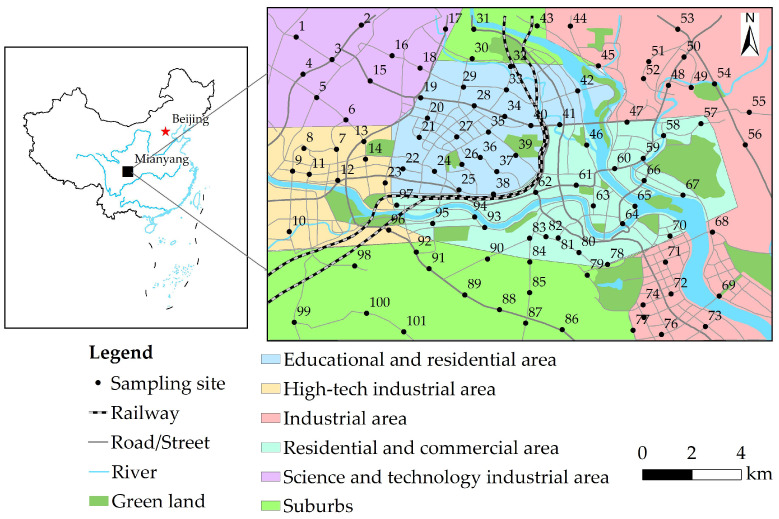
The sampling points in Mianyang urban area, SW China.

According to the principle of uniformity, the representative sampling point layout, and the size of the study area (Figure 1), a 1.70 km × 1.70 km grid was used to presuppose sampling points. During the field sampling process, the actual sampling points were modified according to the sampling feasibility, and there were 101 actual sampling points within the study area. The topsoil samples were collected in August 2021, and the topsoil samples were collected from urban green land and green belt of Mianyang urban area. At each of the sampling points, five sub-samples were placed, and the same amount of soil was collected at the same depth (0–20 cm). After removing rubbish, rocks, and grass roots [25,26], five sub-samples were fully mixed and then put into a numbered polypropylene bag with a weight of about 1.5 kg. The actual location of every topsoil sampling point was noted using global positioning system, and 101 topsoil samples were collected.

All topsoil samples were taken back to the laboratory and air-dried on white porcelain plates, then ground in a mortar and passed through a 1 mm nylon screen; some sieved topsoil samples were taken to determine the physical–chemical properties. About 30 g of the sieved samples were extracted from each sample and ground using vibration of less than 0.075 mm [27]. A 5 g milled soil sample and a plastic ring with an inner diameter of 34 mm were put into the mold. The tablet was pressed using the tablet press under 30-ton pressure, and then the concentrations of 10 HMs (Ba, As, Cr, Co, Pb, Mn, Cu, Ni, Zn, and V) were determined via X-ray fluorescence spectrometer (XRF, Bruker, S8 Tiger, Germany) [20,28]. The standard sample (GSS-2) and 10% repeated samples were used for quality control. The error of measurement was less than 5%.

### 2.3. Data Analysis

#### 2.3.1. Geo-Accumulation Index (*I_geo_*)

*I_geo_*, put forward by Müller in 1969, was generally applied to evaluate HM contamination in topsoil [29,30]. *I_geo_* was calculated using Equation (1) [31]

(1) $$I_{geo}=\log_{2}\frac{C_{i}}{KB_{i}}$$

where *C_i_* is the concentration of HM *i* in the topsoil sample; *B_i_* is the background concentration of HM *i* (in this study, the background content of Sichuan topsoil [32] was used); K is a constant (K = 1.5). Table S1 shows the pollution grade of HMs based on *I_geo_* value.

#### 2.3.2. Potential Ecological Risk Index (*RI*)

*RI*, put forward by Håkanson [33], was computed using the following formula [33]

(2) $$RI=\sum_{i=1}^{n}E_{i}=\sum_{i=1}^{n}\left(T_{i}\times C_{i}/B_{i}\right)$$

where *RI* is the overall ecological risk of all HMs determined in topsoil; *E_i_* is the potential ecological risk index of individual HM *i*; *T_i_* is the toxic response coefficient of HM *i*, reflecting the toxicity degree of HM and the sensitivity of organisms to HM pollution. The toxic response coefficient of As, Co, Cu, Ni, Pb, Cr, V, Ba, Mn, and Zn is 10, 5, 5, 5, 5, 2, 2, 1, 1, and 1, respectively [34]; *C_i_* is the measured content of HM *i*; *B_i_* is the background value of HM *i* [32]. The ecological risk grade based on the value of *RI* and *E_i_* was cited from the literature [35] and is listed in Table S2.

#### 2.3.3. Health Risk Assessment

In this study, the health risk of HMs in urban topsoil to local residents was assessed using the USEPA health risk assessment (HRA) model. Inhabitants are exposed to HM in urban topsoil primarily through direct ingestion, inhalation, and dermal absorption. The 10 HMs studied in this paper all have non-carcinogenic health risks, whereas As, Cr, Co, and Ni also present a carcinogenic risk. Average daily exposure doses (*ADD_ing_*, *ADD_inh_*, *ADD_dermal_*) were calculated using the following Equations (3)–(5) [36]

(3) $$ADD_{ing}=C\times \frac{IngR\times EF\times ED}{BW\times AT}\times 10^{-6}$$

(4) $$ADD_{inh}=C\times \frac{InhR\times EF\times ED}{PEF\times BW\times AT}$$

(5) $$ADD_{dermal}=C\times \frac{SL\times SA\times ABS\times EF\times ED}{BW\times AT}\times 10^{-6}$$

where *ADD_ing_*, *ADD_inh_*, and *ADD_dermal_*, respectively, represent the average daily exposure dose (mg kg^−1^ day^−1^) via direct ingestion, inhalation, and dermal absorption; *C* is the content (mg kg^−1^) of HMs (95% UCL); *IngR* is the ingestion rate, mg day^−1^ [37]; *InhR* is inhalation rate, m^3^ day^−1^ [38]; *EF* means exposure frequency, day year^−1^ [37]; *ED* refers to exposure time, year [37]; *BW* means body weight, kg [39]; *PEF* means the particle emission factor, m^3^ kg^−1^ [37]; *SA* is exposed skin area, cm^2^ [39]; *SL* is skin adhesion, mg cm^−2^ day^−1^ [37]; *ABS* means dermal absorption factor [37]; *AT* is the mean time, day [40,41]. Table S3 shows the values of all parameters.

The non-carcinogenic risk of HMs in urban topsoil is calculated using Equation (6) [36]

(6) $$HI=\sum HQ=\sum \frac{ADD_{ij}}{RfD_{ij}}$$

where *HQ* means the hazard quotient (*HQ*) of HM *i*; *RfD_ij_* means the reference dose of the exposure route *j* of HM *i*; and *HI* means the total non-carcinogenic hazard index of all HMs. The risk is small or negligible if *HI* < 1, and there is a non-carcinogenic risk if *HI* > 1 [36].

Total carcinogenic risk (*TCR*) was computed using Equation (7) [36]

(7) $$TCR=\sum CR=\sum ADD_{ij}\times CSF_{ij}$$

where *CSF_ij_* means the carcinogenic slope factor of the exposure route *j* of HM *i*, (mg kg^−1^ day^−1^)^−1^; *CR* means the carcinogenic risk of individual HMs; *TCR* means the total carcinogenic risk of all HMs. If *TCR* < 10^−6^, the risk is small or negligible; if *TCR* is in the range of 10^−6^–10^−4^, the risk is considered acceptable; if *TCR* > 10^−4^, the carcinogenic risk is high [36]. Table S4 shows the values of *RfD_ij_* and *CSF_ij_* for each exposure route of different HMs.

#### 2.3.4. Monte Carlo Simulation (MCS)

Monte Carlo simulation (MCS) is one of the most effective methods to solve the problem of randomness and uncertainty in risk assessment, which can more truly reflect the risk distribution. The core principle of MCS is that it is based on the law of large numbers and central limit theorem, using large random samples subject to a certain probability distribution model to simulate the possible phenomena [36]. Crystal Ball software, which is one of the most commonly used Monte Carlo modeling tools, is run on Microsoft Excel, and it can combine MCS to predict a particular situation and show the probability of each prediction result. The main steps of risk assessment based on MCS can be summarized as follows: (1) determine the probability distribution model of each HM content according to the measured data; (2) on the premise of obeying a certain distribution model, the random value is simulated according to the distribution of measured data to generate a new random value of HM content; (3) obtain statistical analysis of random output results, generate probability distribution, and carry out quantitative risk assessment. In this study, MCS was used to analyze the uncertainty of the potential ecological risks and human health risks of HMs.

## 3. Results and Discussion

### 3.1. Content of HMs in Topsoil

Table 1 shows the concentrations of 10 HMs in the topsoil of the study area and the reference concentrations of the Sichuan topsoil [32]. The mean content of Co, Ba, Cr, Zn, and Cu was higher than the corresponding reference values of the Sichuan topsoil. The As in 45% of samples, Ba in 86% of samples, Cr in 98% of samples, Co in 44% of samples, Cu in 66% of samples, Ni in 71% of samples, Pb in 23% of samples, Mn in 51% of samples, Zn in 68% of samples, and V in 56% of samples were higher than their corresponding reference values in the Sichuan topsoil. The maximum content of Ba, As, Cr, Co, Cu, Ni, Pb, Mn, Zn, and V in the topsoil was 1.8, 2.9, 3.2, 5.3, 11.9, 1.5, 1.9, 2.1, 3.7, and 1.6 times the reference values in the Sichuan topsoil, respectively. The content of Cu, Mn, Co, As, and Zn varied greatly; their maximums were 22.2, 18.9, 12.7, 10, and 8 times their minimums, respectively.

The larger the values of the standard deviation (SD) and coefficient of variation (CV), the greater the spatial difference of the HMs and the greater the influence of human activities [27,42]. The CV values of the 10 HMs in the Mianyang urban area followed a decreasing order of Cu (91.6%) > Co (62.0%) > Zn (38.2%) > As (37.6%) > Mn (25.5%) > Pb (24.9%) > Cr (23.2%) > Ba (18.9%) > V (16.4%) > Ni (16.2%); in particular, the CV values of Co and Cu were significantly higher than those of the other elements, with both over 60%, suggesting that the spatial distributions of Co and Cu in the topsoil of Mianyang were significantly different and strongly influenced by anthropogenic activities. The CV values of V and Ni were the smallest, showing that their spatial distributions were relatively uniform and less disturbed by human activities.

**Table 1:** The concentrations of HMs (mg kg^−1^) in Mianyang urban area and other cities worldwide, as well as the reference values of Sichuan topsoil.

HMs	As	Ba	Cr	Co	Cu	Ni	Pb	Mn	Zn	V
Mean	11.2	586.8	124.7	20.2	37.8	34.9	28.4	661.5	102.2	96.6
Minimum	3.0	292.3	72.0	7.4	16.7	16.7	16.6	71.6	40.0	42.8
Maximum	30.0	873.4	252.0	93.9	369.9	49.6	60.0	1356.9	320.2	155.5
SD	4.2	110.9	28.9	12.5	34.6	5.6	7.1	168.6	39.0	15.8
CV (%)	37.6	18.9	23.2	62.0	91.6	16.2	24.9	25.5	38.2	16.4
Reference value [32]	10.4	474.0	79.0	17.6	31.1	32.6	30.9	657.0	86.5	96.0
Havana [43]	NA	NA	NA	9.2	10.3	25.6	56.0	NA	104.0	NA
Medak [44]	4.4	NA	244.1	NA	63.6	20.2	24.7	NA	58.8	NA
Vigo [45]	NA	516.8	68.6	NA	66.1	32.0	96.3	531.6	149.0	NA
Ancona [46]	NA	NA	45.6	18.1	63.9	50.9	97.4	NA	199.1	NA
Yan’an [47]	NA	NA	66.2	NA	23.7	37.6	20.2	NA	71.2	NA
Xi’an [48]	NA	NA	81.1	19.3	54.3	34.5	59.7	671.5	186.2	85.2
Ningbo [5]	7.2	NA	80.0	NA	39.9	32.1	51.4	NA	122.6	NA
Shanghai [14]	8.1	NA	101.6	NA	36.7	38.5	38.3	717.6	152.7	NA
Beijing [15]	NA	NA	61.0	NA	31.7	24.0	23.3	NA	92.9	NA
Karamay [19]	20.7	NA	117.9	NA	64.2	42.7	32.6	NA	123.3	NA

SD means standard deviation, CV (%) means coefficient of variation, NA means not available.

In addition, Table 1 shows the average concentration of HMs from other cities worldwide [5,14,15,19,43,44,45,46,47,48]. The concentration of As in Mianyang was higher than in the other cities except for Karamay. The concentration of Ba and V in Mianyang was, respectively, higher than in Vigo and Xi’an. The concentration of Co in Mianyang was higher than in Havana, Ancona, and Xi’an. Except for Medak, the content of Cr in Mianyang was higher than in the other cities. The concentration of Cu was lower than in the other cities except for Havana, Yan’an, Shanghai, and Beijing. The content of Mn was lower than in the other cities except for Vigo. Except for Ancona, Yan’an, Shanghai, and Karamay, the content of Ni was higher than in the other cities. The content of Pb and Zn in the study area was lower than in the other cities except for Medak, Yan’an, and Beijing. As a whole, the average concentration of As, Ba, Cr, Co, Ni, and V in this study was significantly higher than in the other cities, and Mn was at a moderate level, whereas Zn, Pb, and Cu were slightly lower than in the other cities.

By comparing the average concentration of HMs in the urban topsoil of Mianyang and those of the other big cities listed in Table 1, it was found that the content of As in the urban topsoil of Mianyang was higher than in Ningbo and Shanghai. The content of V was higher than in Xi’an. The content of Co was higher than in Havana, Ancona, and Xi’an. The content of Cr was higher than in Ancona, Xi’an, Beijing, Shanghai, and Ningbo. The content of Cu was higher than in Havana, Shanghai, Beijing, but lower than in Ancona, Ningbo and Xi’an. The content of Mn was lower than in Xi’an and Shanghai. The content of Ni was higher than in Havana, Xi’an, Ningbo, and Beijing and lower than in Ancona and Shanghai. The average content of Pb and Zn was higher than in Beijing but lower than in Havana, Ancona, Xi’an, Ningbo, and Shanghai. Compared with the other large cities listed in Table 1, the content of As, V, Co, Cr, and Ni in the urban topsoil of Mianyang City was higher, the content of Cu was at a moderate level, and the content of Mn, Pb, and Zn was lower. This finding indicates that HM pollution in the urban topsoil of emerging medium-sized cities cannot be ignored, and more attention should be paid to it.

In all, the significant diversity of the HM concentrations in the topsoil from different cities might be related to the soil environment, city size, industrial type, urban management level and environmental protection awareness of the urban residents, and the specific reasons need to be deeply investigated and analyzed [49].

### 3.2. Hot Spot Analysis of HMs in Topsoil

**Figure 2:**
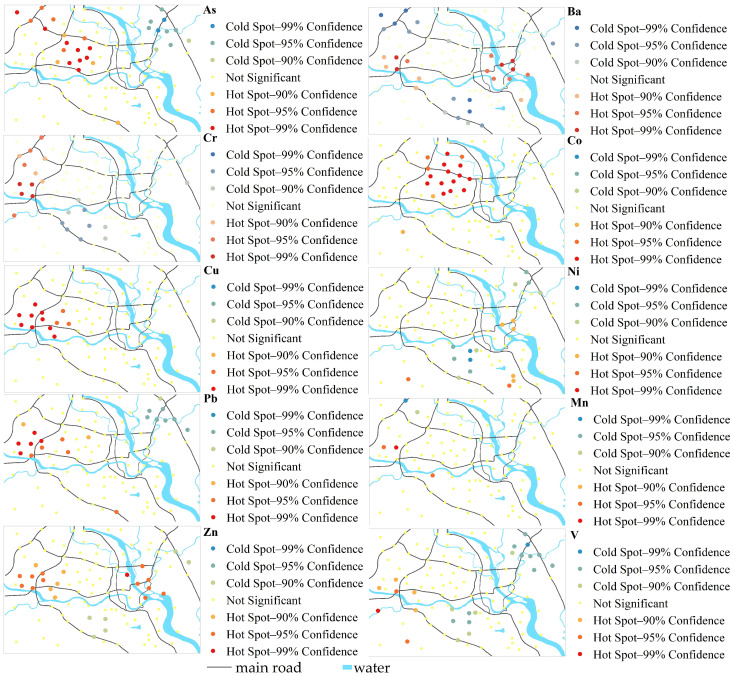
The hot spots of HMs in Mianyang urban area.

Spatial statistics cannot only quantify the concentration of HMs in the aggregated spatial distribution, the size of the intensity in aggregation, and the location and scope of the accumulation area, they can also precisely locate the “Hot spot” and “Cold spot” of the HM content distribution and the level of HM content in the concentration area to provide the basis for determining the priority control of HMs in the study area. Getis-Ord Gi* hot spot analysis was performed on 10 HMs using ArcGIS 10.5 software, and the hot spot maps of 10 HMs were obtained (Figure 2).

Figure 2 shows that the hot spots of As were mainly in the science and technology industrial zone and the educational and residential zone; the hot spots of Ba were mainly concentrated in the high-tech industrial area and the eastern industrial zone; the hot spots of Co were primarily distributed in the educational and residential area; the hot spots of Cr were mainly concentrated in the science and technology industrial zone and the high-tech industrial area; the hot spots of Cu and Mn were mainly concentrated in the high-tech industrial zone; the Ni hot spots were distributed in the eastern industrial zone and the southern suburban area; the Pb hot spots were located in the high-tech industrial area and the educational and residential area; the Zn hot spots were primarily located in the high-tech industrial zone and the eastern industrial zone; and the V hot spots were located in the high-tech industrial zone and the southern suburban zone. Overall, all of the 10 HMs have different spatial aggregation levels, of which Ba, As, Cu, Pb, Co, Cr, and Zn show obvious enrichment trends, but different HMs have different spatial aggregation regions, which may be related to the different sources of HMs.

### 3.3. Results of Geo-Accumulation Assessment

Figure S1 shows the calculated results of *I_geo_* for HMs in the topsoil of Mianyang. The mean *I_geo_* values of HMs show the trend: Cr > Ba > Zn > Cu > Ni > Co > As > V > Mn > Pb. Ba, As, Cr, Co, Pb, Mn, Cu, Ni, Zn, and V presented with an “uncontaminated to moderately contaminated” level in 14, 13, 49.5, 8, 3, 5, 4, 1, 8, and 1% of samples, respectively. Cr, Co, Cu, and Zn exhibited a “moderately contaminated” level in 1, 3, 1, and 2% of samples, respectively. For Cu, 1% of samples were at a “moderately to heavily contaminated” level.

**Figure 3:**
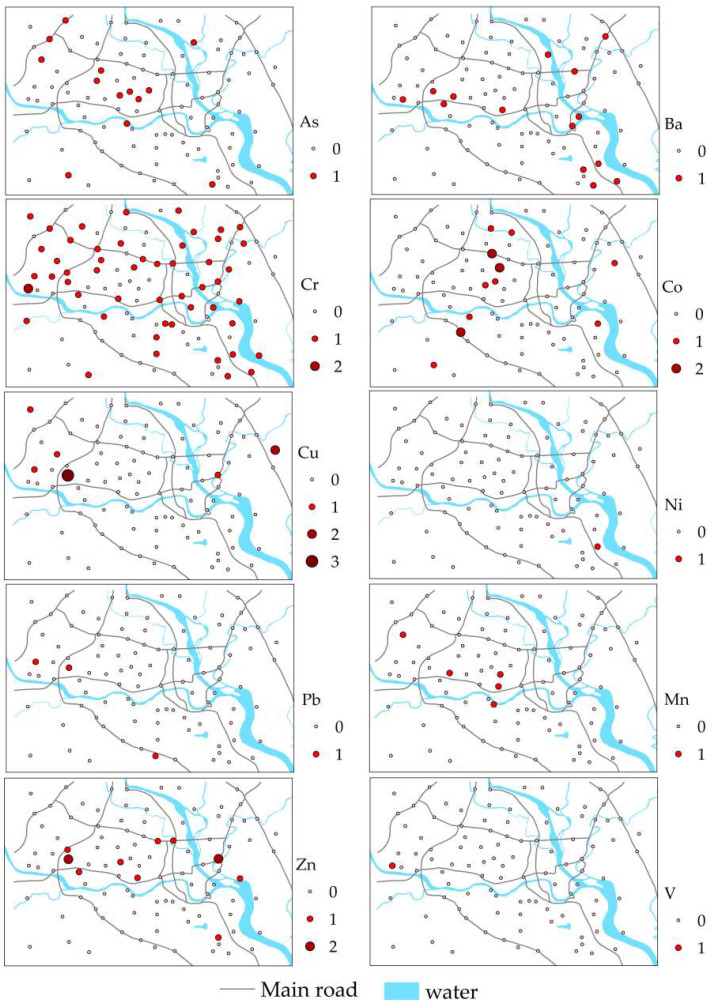
The *I_geo_* level of HMs in Mianyang urban area.

Figure 3 shows the spatial distribution of the HM pollution level in the study area based on the *I_geo_* value. The samples with As pollution were mainly concentrated in the northwest science and technology industrial zone and the central comprehensive educational and residential area. The samples with Ba pollution were mainly concentrated in the western high-tech industrial area and the eastern industrial area, which was possibly due to the alloy, paint, ceramic, and glass manufacturing [50]. The Cr pollution samples were mainly concentrated in the northwest science and technology industrial zone, the western high-tech industrial area, and the eastern industrial area. The main industries in these areas are machinery factories, mechanical and electrical factories, and plastic and steel door and window factories. This result is in line with previous research, i.e., Cr is wildly used in the production of alloy, stainless steel, automobile parts [51].

The samples with Co pollution were mainly concentrated in the educational and residential area, a newly developed area where the soil environment was severely affected by human activities. The Cu pollution samples mainly appeared in the high-tech industrial zone. The accumulation of Cu in the area might be attributed to metal casting and machinery production; this is consistent with previous research findings that Cu originates from metal casting and machine parts manufacturing [52]. The samples with Ni and V pollution were both found along the river. The Ni pollution samples were in the east, near the Fujiang River, and the V pollution samples were in the west, near the Anchang River. The samples with Pb pollution were distributed along the urban trunk roads, which may be caused by auto manufacturing and vehicle emissions. Previous studies have also found that Pb is often used in brass automotive radiators and car lubricants [52]. The Mn pollution samples were located in the western high-tech industrial area, which may be caused by anthropogenic activity such as the chemical industry and smelting [53]. The Zn pollution samples mainly appeared in the high-tech industrial zone and the eastern industrial zone of the Mianyang urban area, which may be due to the production process of automobile tires, zinc alloys, and galvanizing. This finding is consistent with previous studies; that is, industrial waste and car body wear lead to an increase in Zn content in the soil [54,55].

### 3.4. Results of Potential Ecological Risk Assessment

#### 3.4.1. Potential Ecological Risk Factor of Individual HMs (*E_i_*) and Potential Ecological Risk Index (*RI*) of All HMs

Figure S2 shows the assessment results of the potential ecological risk of HMs in topsoil from the Mianyang urban area. The *E_i_* values of Ba, Cr, Ni, Pb, Mn, Zn, and V in all samples and that of As, Co, and Cu in most topsoil samples were less than 15, showing a low ecological risk. Whereas the 14% *E_i_* value of As, 3% *E_i_* value of Co, and 1% *E_i_* value of Cu were between 15 and 30, indicating a moderate ecological risk, the 1% *E_i_* value of Cu was in the range of 30–60, indicating a considerable ecological risk.

**Figure 4:**
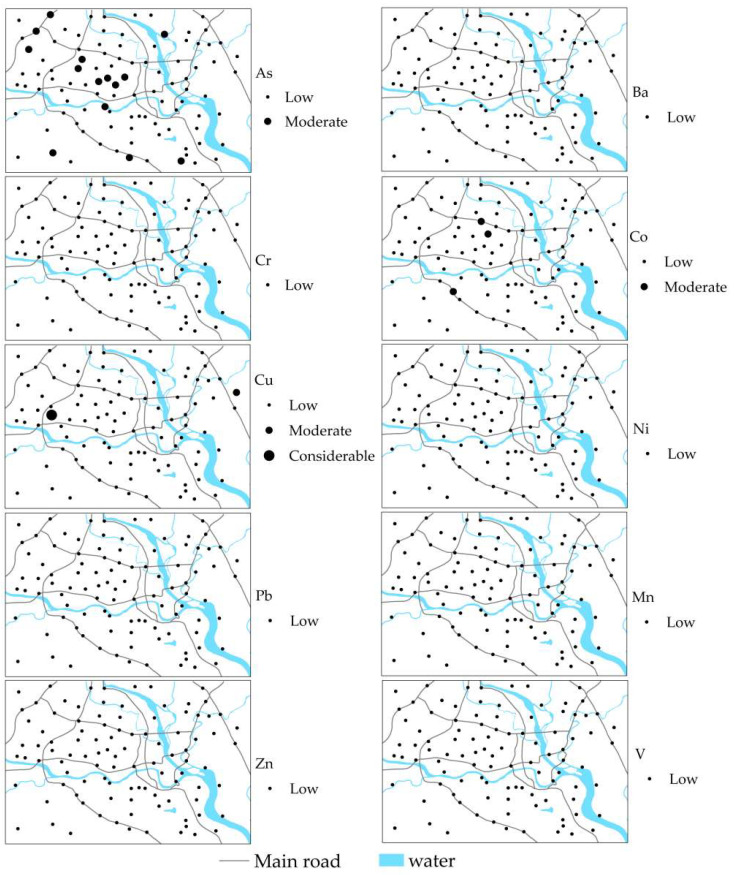
The *E_i_* degree of HMs in Mianyang urban area.

Figure 4 shows the ecological risk degrees of HMs in the Mianyang urban area. The samples with an As moderate ecological risk were mainly distributed in the science and technology industrial zone and the central comprehensive educational and residential area. Two samples with a Co moderate ecological risk were distributed in the educational and residential zone, and one sample was found in the high-tech industrial zone. One sample with a Cu moderate ecological risk was found in the eastern industrial area, and one sample with a considerable ecological risk appeared in the high-tech industrial zone.

**Figure 5:**
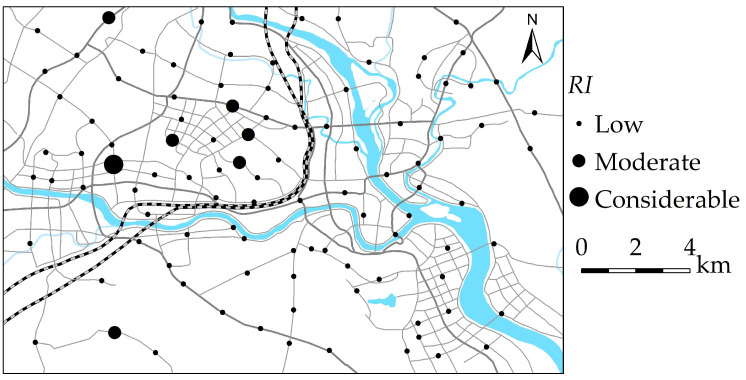
The *RI* degree of HMs in Mianyang urban area.

The *RI* values of HMs in the topsoil samples were between 22.8 and 105.2, with an average value of 41.1. The HMs in 93% of the topsoil samples exhibited a low ecological risk, and 7% of the topsoil samples posed a moderate and considerable ecological risk. Figure 5 shows the *RI* degree of HMs in the topsoil of Mianyang based on the *RI* values. The moderate and considerable ecological risk samples were mainly located in the central educational and residential region, the science and technology industrial zone, and the western high-tech industrial area.

#### 3.4.2. Assessment of Potential Ecological Risk Based on MCS

The content of HMs was taken as uncertainty parameters, and their distribution characteristics were analyzed using Crystal Ball software [56]; the fitting results are shown in Table S5. Calculated according to Equation (2) and with *RI* taken as the target variable, the *RI* histogram (Figure S3) was obtained using MCS with Crystal Ball software [56]; the predicted results belonged to Student’s t distribution, and the confidence interval at a 95% level of *RI* was (37.7, 46.7). The simulation results of *RI* (42.1) were basically consistent with the deterministic calculation results (41.1), which also showed that the HM content after simulation was basically consistent with the measured data, indicating that the simulation results were credible, and the potential ecological risk of HMs showed a low ecological risk in the study area. The sensitivity analysis figure (Figure S3) of *RI* was obtained using the sensitivity analysis of the *RI* predictive value; Figure S3 shows that the sensitivity coefficients of As, Cu, Pb, Co, Cr, and V were 74.1, 7.5, 6.3, 5.8, 2.5, and 1.7%, respectively, indicating that As was the main contributor to the potential ecological risk. Cu, Pb, Co, Cr, and V also played a role. Comparing the evaluation results of the *I_geo_* and *RI* methods, it was found that the evaluation results were generally consistent, but there were still some differences. For example, Cr was assessed as being at an “uncontaminated to moderately contaminated” level by *I_geo_* and as a low ecological risk by *RI*; this might be caused by the different toxicities of HMs. The *I_geo_* method focuses on the topsoil background values and single HM content, whereas the *RI* method also takes into account the toxicity and integrated effects of several HMs, and the sensitivity of each HM element in *RI* was related to the content of HM and the biological toxicity coefficient of the HM.

### 3.5. Concentration-Oriented Human Health Risk Assessment

#### 3.5.1. Human Health Risk Assessment

**Figure 6:**
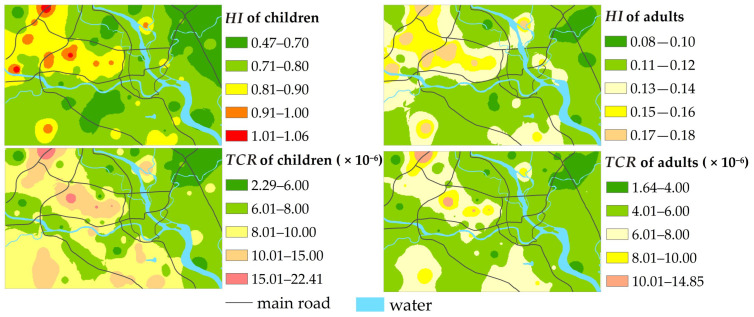
*HI* and *TCR* for both children and adults.

Figure 6 shows the spatial distribution of the non-carcinogenic risk and carcinogenic risk of HMs in topsoil from the Mianyang urban area. The non-carcinogenic risk for children was higher than 1 in three sampling points located in the science and technology industrial zone, the high-tech industrial zone, and the educational and residential zone, respectively, indicating these areas presented a non-carcinogenic risk, and some sampling points had *HI* values close to 1, showing a great tendency toward non-carcinogenic risk, whereas the adults’ non-carcinogenic risk was within a safe range in all sampling sites. The total carcinogenic risk (*TCR*) for both children and adults was within an acceptable range at all sampling sites. Although the non-carcinogenic risks of most sampling sites were negligible and the carcinogenic risks of all sampling sites were within the acceptable range, the values of some sampling sites were high. The value of *HI* and *TCR* showed a strong spatial consistency; that is, *HI* and *TCR* for both children and adults were higher in the science and technology industrial zone, the high-tech industrial zone, and the educational and residential zone than in other zones, posing a threat to the health of the local residents in the three zones. Therefore, the harm of HMs in the topsoil to human health cannot be ignored, and further risk control should be particularly considered, and remedial action should be taken.

Table 2 shows the *HQ* and *HI* of HMs for both children and adults. The *HQ* values of the HMs for children and adults due to the three exposure ways followed the order of *HQ_ing_* > *HQ_dermal_* > *HQ_inh_*, indicating that direct ingestion was the major route for residents exposed to HMs in Mianyang urban topsoil. By comparing the *HQ* values of the HMs for children and adults through different exposure routes, it was found that the *HQ_ing_*, *HQ_inh_*, and *HQ_dermal_* values of the 10 HMs for children were all higher than those for adults, indicating that children had a greater non-carcinogenic risk than adults; therefore, the prevention and control of HM pollution should be strengthened in urban topsoil environments, and the health protection of children should be paid attention to. The *HQ* values of 10 HMs for children were also higher than that for adults. For children, the order of *HQ* values was Cr > As > V > Mn > Ba > Pb > Ni > Co > Cu > Zn; whereas for adults, the *HQ* values were in the order of Cr > As > Mn > V > Ba > Pb > Ni > Co > Cu > Zn. The *HQ* value of Cr and As was 2–149 times higher than that of other HMs, indicating that the control of these HMs (Cr and As) should be strengthened.

The *CR* values of HMs for both children and adults followed the order of As > Cr > Co > Ni. The *CR* values of Co, Cr, and Ni were < 10^−6^ (Table 2), showing that the cancer risks due to these HMs were negligible, and the *CR* value of As was within the acceptable range (10^−6^–10^−4^), meaning that As presented a greater carcinogenic risk than Cr, Co, and Ni. By comparing the *CR* for children and adults, it was found that the *CR* of As for children was higher than that for adults, whereas the *CR* values of Cr, Co, and Ni for children were lower than that for adults. In general, the *TCR* for children was higher than that for adults; children were at a greater carcinogenic risk than adults, and As contributed the most to the carcinogenic risk for both children and adults, followed by Cr, which should be paid more attention. 

#### 3.5.2. Assessment of Human Health Risk Based on MCS

The content of 10 HMs in topsoil were selected as the uncertainty parameters, and their distribution characteristics were analyzed using Crystal Ball software (Table S5). The evaluation model was established according to Equations (3)–(7), and MCS was performed with Crystal Ball software [56]; the probability ranges and sensitivity analyses for the non-carcinogenic and carcinogenic risks of HMs were obtained (Figures S4 and S5). The results show that the confidence level of 95% values of the children’s non-carcinogenic risk, the adults’ non-carcinogenic risk, the children’s carcinogenic risk, and the adults’ carcinogenic risk were (0.7, 0.9), (0.1, 0.2), (5.1 × 10^−6^, 1.1 × 10^−5^), and (3.5 × 10^−6^, 7.5 × 10^−6^), respectively. The stochastic simulation results of the non-carcinogenic risk and the carcinogenic risk of HMs were basically consistent with those of the deterministic calculation (Table S6), indicating that the simulation results were credible.

By analyzing the sensitivity of the uncertainty parameters, we found that Cr and As in 10 HMs have a significant impact on the non-carcinogenic risk for children and adults: As and Cr contribute 55.8 and 30.2%, respectively, to the non-carcinogenic risk for children; As and Cr account for 53.5 and 27.6% of the non-carcinogenic risk for adults, respectively. The non-carcinogenic risk contribution of As and Cr suggests that strengthening the control and management of As and Cr in topsoil is very important for reducing the non-carcinogenic risk of HMs in the Mianyang urban area. The contribution of As to children’s carcinogenic risk and adults’ carcinogenic risk is nearly 100 and 99.9%, respectively, and Cr, Co, and Ni have little effect on both the children’s carcinogenic risk and adults’ carcinogenic risk. Through the analysis of carcinogenic risk sensitivity, we found that the contribution of As content in topsoil to the carcinogenic risk is relatively high due to the high carcinogenic toxicity coefficient of As. So, we conclude that reducing the As content in topsoil is an effective method to reduce the carcinogenic risk.

### 3.6. Source-Oriented Human Health Risk Assessment

A source-oriented human HRA is more significant to target environmental management than a single source appointment or HRA. Quantifying the contribution of HMs from different sources to the human health risk can rank topsoil HMs that need priority prevention and control [57] and select the HM with the greater impact for priority management and control to minimize the risk to human beings. Using the PMF-HRA model [36], according to the source appointment results of the PMF model, i.e., our previous research [20], non-carcinogenic risk and carcinogenic risk were evaluated using the contribution rates of different sources. The contribution of non-carcinogenic risks from different sources to children and adults are more similar; the results are consistent with those of Huang et al. [36]. Of the three pollution sources of health risk factors, the mixed source of industry and traffic is the main non-carcinogenic risk source, followed by natural sources and industrial sources. The contribution of single HMs from the mixed source to the total non-carcinogenic health risk for children was in the order of Cr (21.53%) > As (7.49%) > Mn (4.28%) > V (3.74%) > Ba (2.49%) > Pb (2.33%) > Ni (0.36%) > Cu (0.24%) > Co (0.20%) > Zn (0.09%), and the contribution of an individual HM from the mixed source to the total non-carcinogenic risk for adults was in the order of Cr (20.79%) > As (7.26%) > Mn (5.31%) > V (3.68%) > Ba (2.44%) > Pb (2.15%) > Ni (0.33%) > Co (0.25%) > Cu (0.22%) > Zn (0.08%). On the whole, Cr and As contribute more to the non-carcinogenic risks for children and adults.

The mixed source is the major anthropogenic source that influences the carcinogenic risk. The contribution of single HMs from the mixed source to the total carcinogenic risk for children was in the order of As (24.79%) > Cr (0.49%) > Co (0.01%) > Ni (1.24 × 10^−3^%); and the contribution of individual HMs from the mixed source to the adults’ total carcinogenic risk was in the order of As (24.07%) > Cr (2.36%) > Co (0.04%) > Ni (0.01%). Overall, As contributes the most to the total carcinogenic risk for children and adults, followed by Cr.

Our study found that children are more sensitive than adults when exposed to HMs in the topsoil, which is reflected in the fact that children show higher average *HI* and *TCR* values than adults; this is largely due to their direct ingestion [36,58,59]. Therefore, the prevention and control of health risks in the Mianyang urban area should pay special attention to children’s exposure to HMs.

By analyzing the sources of pollutants, it was found that 66.93% of Cr and 24.98% of As came from the mixed source; therefore, the mixed source of industry and traffic was identified as the priority anthropogenic source of pollution control. Figures S4 and S5 show that the contribution of Cr and As was greater both in terms of non-carcinogenic risk and carcinogenic risk, thus these two HM elements were identified as priority pollutants for human health risk control. The high concentration sampling sites of Cr and As were mainly concentrated in the northwest science and technology industrial zone, the western high-tech industrial area, and the central science and technology industrial zone (Figure 2). These areas are newly developed regions with many factories and enterprises, a large traffic volume, and a dense population, where human activities have greatly disturbed the original environment. Combining the results of the hot spot analysis, *I_geo_*, *E_i_*, *RI*, *HI*, and *TCR*, we found that the educational and residential region, the science and technology industrial zone, and the high-tech industrial area in Mianyang City are areas with high HM pollution levels, ecological risks, non-carcinogenic risks, and carcinogenic risks and areas with HM pollution priority.

## 4. Conclusions

Elevated Ba, Cr, Cu, and Zn were found in the topsoil of the Mianyang urban area. HMs analyzed in the topsoil exhibited uncontaminated levels and low ecological risks in most samples. The overall ecological risk of HMs was low in 93% of samples, whereas it was moderate to considerable in 7% of samples. As was a major contributor to the overall ecological risk. The investigated HMs presented no significant non-carcinogenic hazard to local adult residents, but there was a non-carcinogenic hazard to children in 3% of the sampling sites, which should be noticed by the relevant departments. The carcinogenic risks of As, Cr, Co, and Ni were acceptable. The mixed source was the main anthropogenic contributor to the non-carcinogenic and carcinogenic risks of HMs. Cr and As were the priority HMs, and the mixed source was the priority source. The educational and residential region, the science and technology industrial zone, and the high-tech industrial area were the priority areas for HM pollution control. Due to the limitation of the XRF, Cd was not studied in this work; this will be performed in future research work.

It is suggested that the risk control of soil pollution should focus on children’s susceptibility and the high risk of Cr and As and strengthening the control of anthropogenic pollution sources to effectively protect the health of local residents.

## Figures and Tables

**Table 2:** Non-carcinogenic/carcinogenic risk index of HMs in Mianyang urban topsoil.

Element		As	Ba	Cr	Co	Cu	Ni	Pb	Mn	Zn	V	10 HMs
Child	*HQ_ing_*	1.86 × 10^−1^	4.31 × 10^−2^	2.10 × 10^−1^	4.67 × 10^−3^	4.32 × 10^−3^	9.04 × 10^−3^	4.07 × 10^−2^	7.31 × 10^−2^	1.67 × 10^−3^	7.11 × 10^−2^	
	*HQ_inh_*	5.17 × 10^−6^	5.89 × 10^−4^	6.17 × 10^−4^	4.57 × 10^−4^	1.20 × 10^−7^	2.45 × 10^−7^	1.13 × 10^−6^	6.57 × 10^−3^	4.66 × 10^−8^	1.99 × 10^−6^	
	*HQ_dermal_*	3.87 × 10^−2^	1.75 × 10^−3^	3.00 × 10^−2^	1.66 × 10^−5^	4.10 × 10^−5^	9.54 × 10^−5^	7.74 × 10^−4^	5.21 × 10^−3^	2.38 × 10^−5^	2.03 × 10^−2^	
	*HQ*	2.25 × 10^−1^	4.54 × 10^−2^	2.41 × 10^−1^	5.14 × 10^−3^	4.36 × 10^−3^	9.14 × 10^−3^	4.15 × 10^−2^	8.49 × 10^−2^	1.69 × 10^−3^	9.14 × 10^−2^	
	*HI*											7.49 × 10^−1^
Adult	*HQ_ing_*	2.89 × 10^−2^	6.69 × 10^−3^	3.27 × 10^−2^	7.25 × 10^−4^	6.71 × 10^−4^	1.40 × 10^−3^	6.33 × 10^−3^	1.14 × 10^−2^	2.59 × 10^−4^	1.10 × 10^−2^	
	*HQ_inh_*	4.23 × 10^−6^	4.82 × 10^−4^	5.04 × 10^−4^	3.73 × 10^−4^	9.82 × 10^−8^	2.01 × 10^−7^	9.25 × 10^−7^	5.38 × 10^−3^	3.81 × 10^−8^	1.62 × 10^−6^	
	*HQ_dermal_*	7.94 × 10^−3^	3.60 × 10^−4^	6.15 × 10^−3^	3.41 × 10^−6^	8.41 × 10^−6^	1.96 × 10^−5^	1.59 × 10^−4^	1.07 × 10^−3^	4.88 × 10^−6^	4.16 × 10^−3^	
	*HQ*	3.68 × 10^−2^	7.53 × 10^−3^	3.93 × 10^−2^	1.10 × 10^−3^	6.79 × 10^−4^	1.42 × 10^−3^	6.49 × 10^−3^	1.78 × 10^−2^	2.64 × 10^−4^	1.52 × 10^−2^	
	*HI*											1.27 × 10^−1^
Child	*CR_ing_*	6.69 × 10^−6^										
	*CR_inh_*	1.88 × 10^−9^		5.93 × 10^−8^	2.04 × 10^−9^		3.40 × 10^−10^					
	*CR_dermal_*	1.39 × 10^−6^										
	*CR*	8.08 × 10^−6^		5.93 × 10^−8^	2.04 × 10^−9^		3.40 × 10^−10^					
	*TCR*											8.15 × 10^−6^
Adult	*CR_ing_*	4.16 × 10^−6^										
	*CR_inh_*	6.15 × 10^−9^		1.94 × 10^−7^	6.69 × 10^−9^		1.11 × 10^−9^					
	*CR_dermal_*	1.14 × 10^−6^										
	*CR*	5.31 × 10^−6^		1.94 × 10^−7^	6.69 × 10^−9^		1.11 × 10^−9^					
	*TCR*											5.51 × 10^−6^

## Data Availability

The datasets utilized in this study are available upon request from the corresponding author.

## Supplementary Materials

The following supporting information can be downloaded at: https://www.mdpi.com/article/10.3390/ijerph192215126/s1, Figure S1: Box plots of *I*_geo_ for HMs in Mianyang urban topsoil samples; Figure S2: Box plots of *E_i_* for HMs in Mianyang urban topsoil samples; Figure S3: Probability distribution and sensitivity analysis of potential ecological risk index (*RI*); Figure S4: Probability distribution and sensitivity analysis of HM non-carcinogenic risk; Figure S5: Probability distribution and sensitivity analysis of HM carcinogenic risk; Table S1: Pollution degree and *I_geo_* value; Table S2: Grade of potential ecological risk index; Table S3: Parameter value of exposure assessment model; Table S4: Parameter value of exposure assessment model; Table S5: Distribution test and fitting results of HM contents; Table S6: The mean value of the simulation results and the mean value of the deterministic calculation results of the non-carcinogenic/carcinogenic risk of HMs.
