# Supplementary material for: Contamination and Probabilistic Ecological–Health Risk of Heavy Metal(loid)s in Urban Topsoil of Mianyang, SW China
Source: Int J Environ Res Public Health. 2022 Nov 16;19(22):15126. doi: 10.3390/ijerph192215126 (PMC9691029; doi:10.3390/ijerph192215126)
Supplement: Supplementary file 1 [file ijerph-19-15126-s001.zip › ijerph-1981025-supplementary.pdf]

Supplementary Materials

Contamination and Probabilistic Ecological-Health Risk of Heavy Metal(Loid)S in Urban Topsoil of Mianyang, SW China

Huaming Du [1,2] and Xinwei Lu [2,*]

[1] School of Resource and Environment Engineering, Mianyang Normal University, Mianyang 621000, China
[2] Department of Environmental Science, School of Geography and Tourism, Shaanxi Normal University, Xi'an 710119, China
* Correspondence: luxinwei@snnu.edu.cn

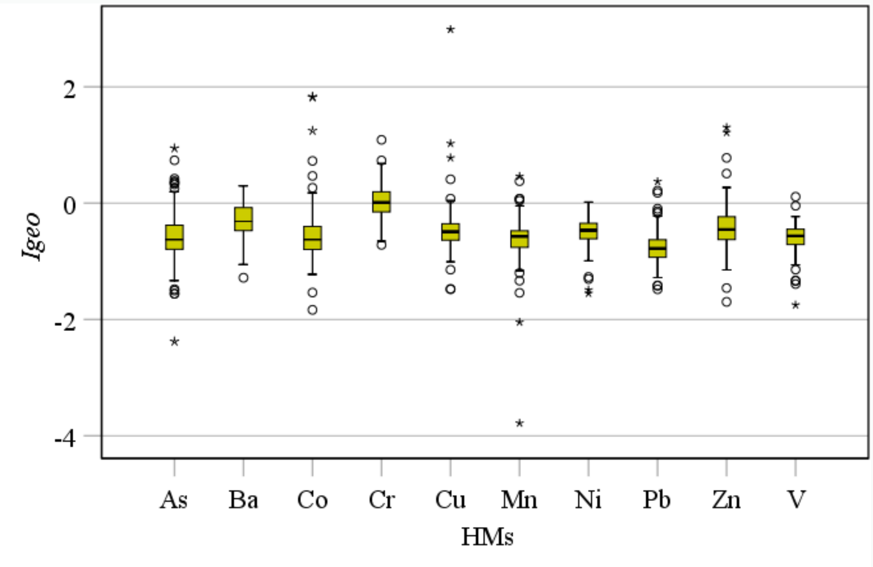

Figure S1. Box-plots of I_{geo} for HMs in Mianyang urban topsoil samples.

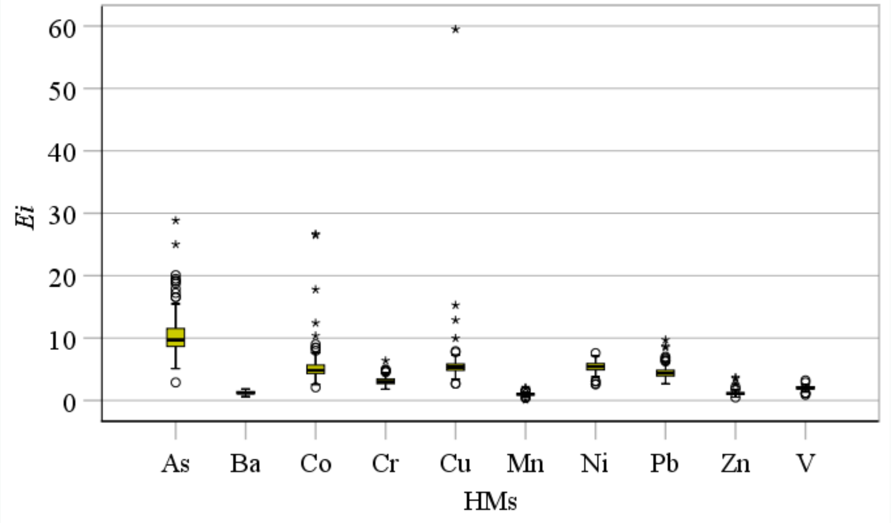

Figure S2. Box-plots of E_i for HMs in Mianyang urban topsoil samples.

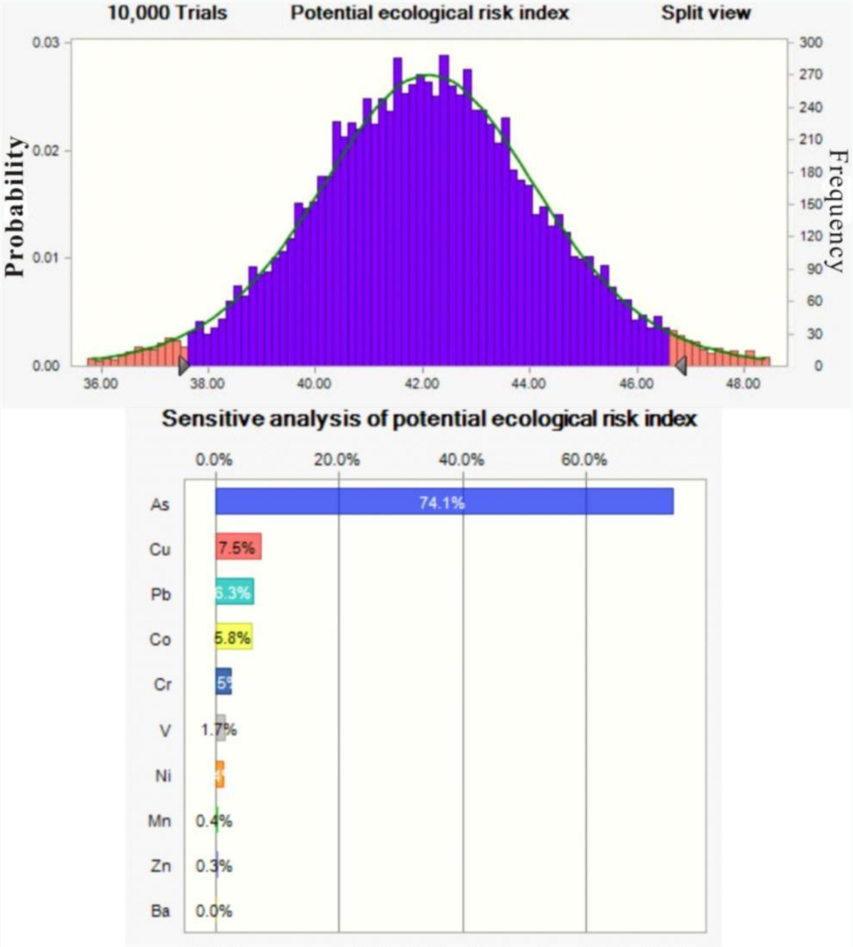

Figure S3. Probability distribution and sensitivity analysis of potential ecological risk index (*RI*).

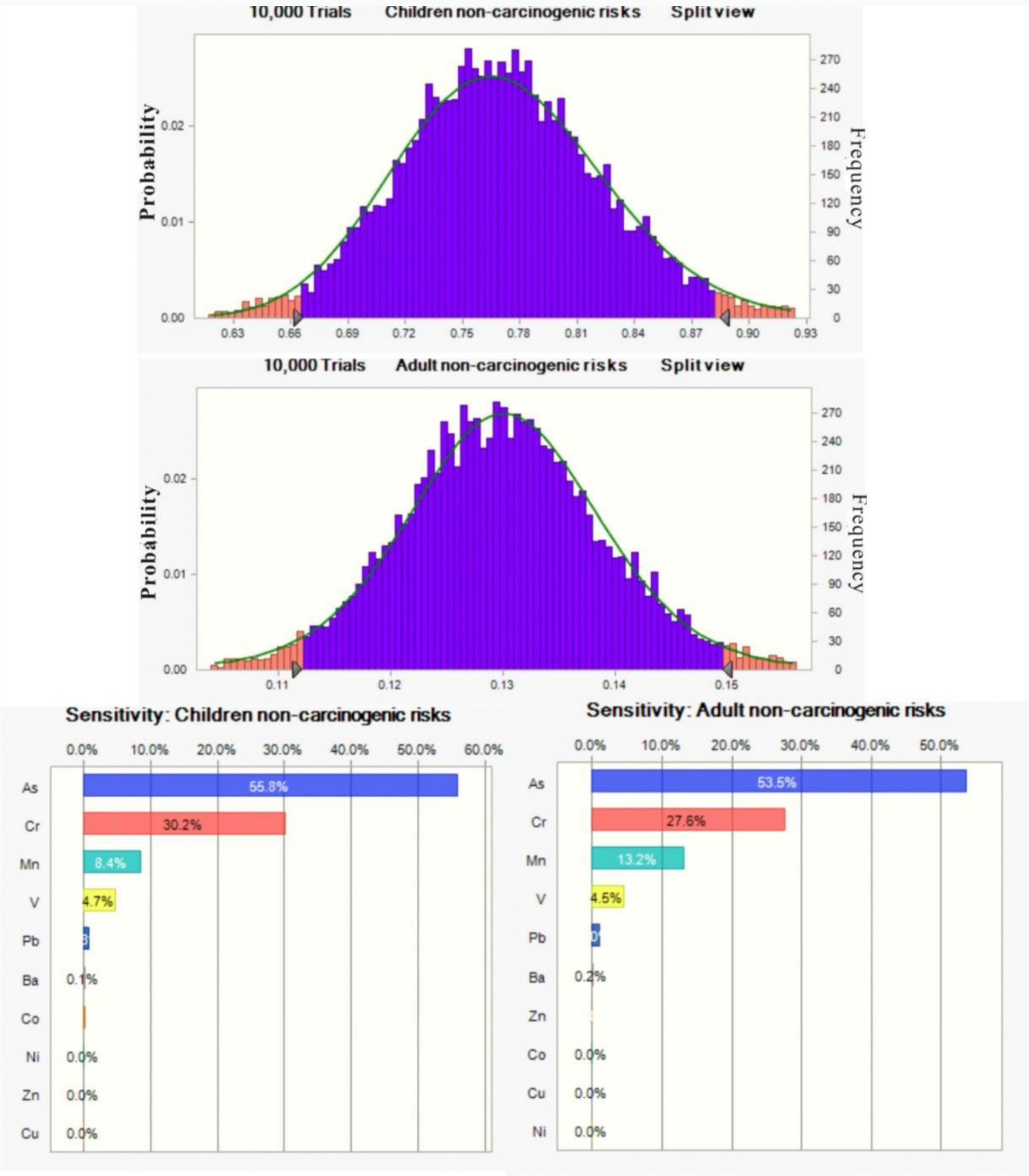

Figure S4. Probability distribution and sensitivity analysis of HM non-carcinogenic risk.

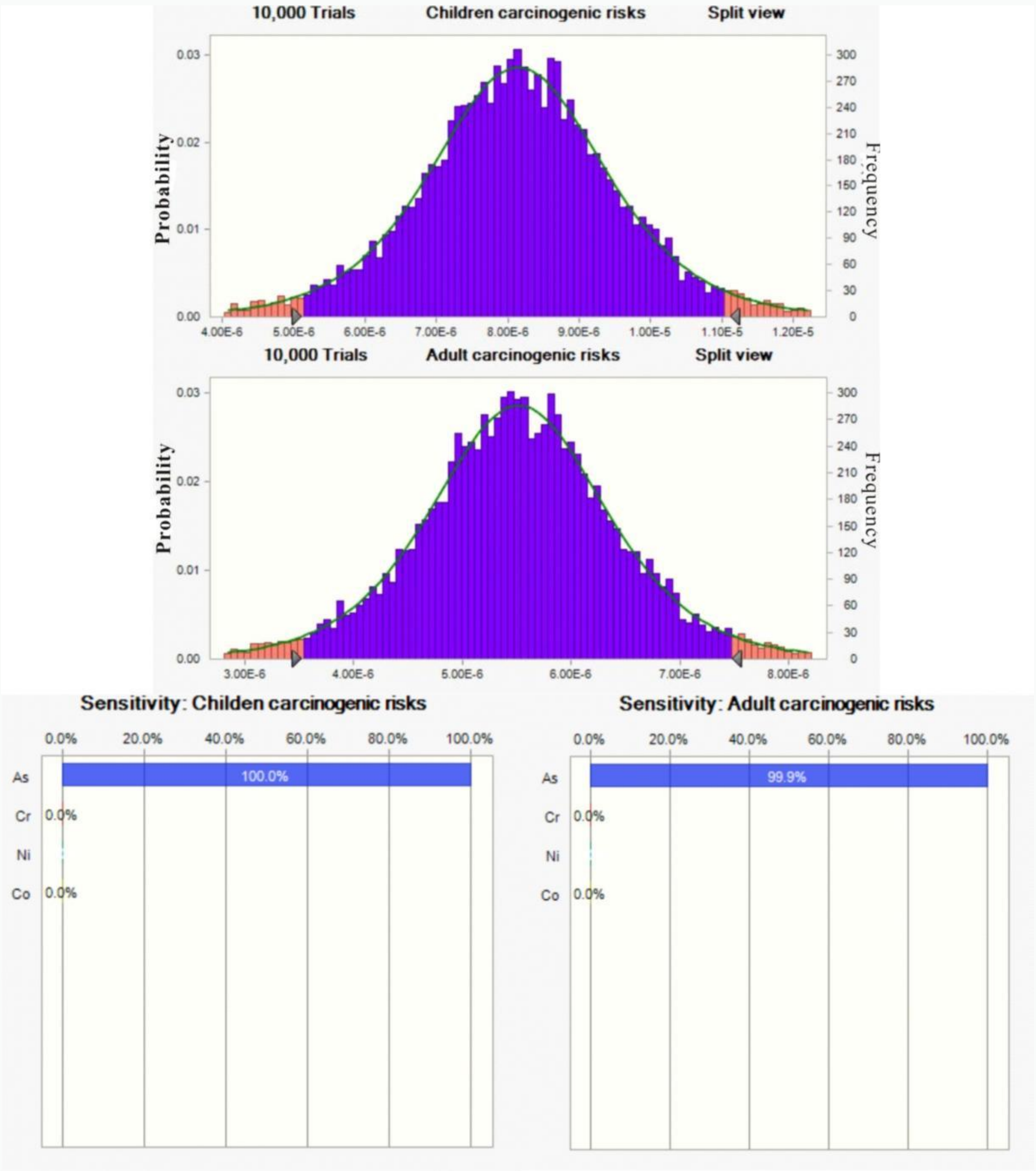

Figure S5. Probability distribution and sensitivity analysis of HM carcinogenic risk.

Table S1. Pollution degree and I_{geo} value.

I_{geo}	Grade	Pollution degree
≤0	0	Uncontaminated
0-1	1	Uncontaminated to moderately contaminated
1-2	2	Moderately contaminated
2-3	3	Moderately to heavily contaminated
3-4	4	Heavily contaminated
4-5	5	Heavily to extremely contaminated
≥5	6	Extremely contaminated

Table S2. Grade of potential ecological risk index.

E_i	*RI*	Ecological risk degree
<15	<50	Low
15-30	50-100	Moderate
30-60	100-200	Considerable
60-120	≥200	High
≥120		Very high

Table S3. Parameter value of exposure assessment model.

Parameter	Meaning	Unit	Child	Adult	Literature
C	Concentration of HMs	mg kg^{-1}	95% UCL	95% UCL	This work
ED	Exposure duration	year	6	24	[37]
BW	Average body weight	kg	19.2	61.8	[39]
EF	Exposure frequency	day $year^{-1}$	180	180	[37]
AT(non-carcinogens)	Average time	day	365×ED	365×ED	[40]
AT(carcinogens)	Average time	day	365×75	365×75	[40,41]
IngR	Ingestion rate	mg day^{-1}	200	100	[37]
InhR	Inhalation rate	m^3 day^{-1}	7.6	20	[38]
PEF	Particle emission factor	m^3 kg^{-1}	1.36×10^9	1.36×10^9	[37]
SL	Skin adherence factor	mg cm^{-2} day^{-1}	0.2	0.07	[37]
SA	Exposed skin area	cm	2848	5374	[39]
ABS	Dermal absorption factor		0.001 (0.03 for As)		[37]

Table S4. Parameter value of exposure assessment models.

Element	As	Ba	Cr	Co	Cu	Ni	Pb	Mn	Zn	V
RfD_{ing}	3.00E-04	7.00E-02	3.00E-03	2.00E-02	4.00E-02	2.00E-02	3.50E-03	4.60E-02	3.00E-01	7.00E-03
RfD_{inh}	3.01E-04	1.43E-04	2.86E-05	5.71E-06	4.02E-02	2.06E-02	2.52E-03	1.43E-05	3.00E-01	7.00E-03
RfD_{dermal}	1.23E-04	4.90E-03	6.00E-05	1.60E-02	1.20E-02	5.40E-03	5.25E-04	1.84E-03	6.00E-02	7.00E-05
CSF_{ing}	1.50E+00									
CSF_{inh}	1.51E+01		4.20E+01	9.8E+00		8.40E-01				
CSF_{dermal}	3.66E+00									

Table S5. Distribution test and fitting results of HM contents.

HMs	Mean	Median	Standard deviation	Minimum	Maximum	Distribution
As	11.18	10.1	4.20	3	30	Logistic
Ba	586.84	572.5	110.90	292.6	873.4	Weibull
Cr	124.65	119.3	28.90	72	252	Max Extreme
Co	20.15	17.1	12.50	7.4	93.9	Lognormal
Cu	37.81	33.2	34.60	16.7	369.9	Lognormal
Ni	34.90	35.4	5.70	16.7	49.6	Weibull
Pb	28.41	27	7.10	16.6	60	Max Extreme
Mn	661.45	663.3	168.60	71.6	1356.9	Logistic
Zn	102.21	87.4	39.00	40	320.2	Lognormal
V	96.58	97.4	15.80	42.8	155.5	Student's t

Table S6. The mean value of the simulation results and the mean value of the deterministic calculation results of the non-carcinogenic/carcinogenic risk of HMs.

	The stochastic simulation results		The deterministic calculation results	
	non-carcinogenic risk	carcinogenic risk	non-carcinogenic risk	carcinogenic risk
Child	7.70E-01	8.17E-06	7.49E-01	8.15E-06
Adult	1.30E-01	5.53E-06	1.27E-01	5.51E-06
